# Effect of the Partial Substitution of NaCl with L-Arg on the Gel Properties and Aggregation Behavior of Beef Myosin

**DOI:** 10.3390/foods14040680

**Published:** 2025-02-17

**Authors:** Chuanlong Yu, Lingli Chen, Kehui Ouyang, Hui Chen, Suyun Lin, Wenjun Wang

**Affiliations:** 1 College of Food Science and Engineering, Jiangxi Agricultural University, Nanchang 330045, China; yuchuanlongvip@163.com (C.Y.);; 2 College of Animal Science and Technology, Jiangxi Agricultural University, Nanchang 330045, China

**Keywords:** myosin, L-Arg, aggregation behavior, salt reduction

## Abstract

With 0.3 M KCl replacing NaCl, the effect of L-Arg on beef myosin’s gel, structure, and aggregation was investigated. L-Arg could enhance hydrogen and ionic bonds, increasing myosin solubility and reducing turbidity. The content of regular secondary structures (β-sheet) increased, and the content of random coil structures decreased significantly (*p* < 0.05). The results of dynamic light scattering (DLS) and atomic force microscope (AFM) further demonstrated that L-Arg could improve the uniformity and dispersion of myosin aggregate size and inhibit the aggregation of myosin between the head and the tail. Moreover, as the hydrophobic interaction and disulfide bonds were the main forces, a thermal gel dominated by myosin oligomers and filaments was formed. In the 0.3 M KCl, 0.1 M NaCl, and 0.2 M L-Arg system, the hardness, elasticity, and water holding capacity (WHC) of beef myosin gel were effectively improved, providing a salt reduction reference for beef products.

## 1. Introduction

Beef protein, a prime protein source, is essential for human physiological functions and growth. Beef gel products like patties and meatballs are favored for their unique texture, a result of specific protein interactions during gelation, influenced by amino acid profiles and processing conditions. Myosin, the main component of myofibrillar proteins (MPs), represents a key contribution to the gelation [1]. Thermal gelation, triggered by myosin denaturation, facilitates continuous frame formation through intermolecular covalent and non-covalent interactions [2]. In our previous work, it was found that 0.6 M NaCl was employed for the complete dissolution of MPs at pH 6.0, leading to the formation of superior gels [3]. Noticeably, excessive NaCl intake poses a threat to human health [4]. Thus, attempts have been made to enhance myosin gel properties under conditions of limited sodium content. By means of enhancing the hydrophobic interaction and disulfide bond of myosin, KCl partially replaces NaCl (0.3 M KCl + 0.3 M NaCl) and could promote myosin cross-linking to form smooth gels in our previous study [5]. However, excessive KCl addition may lead to sensory defects in the product [6].

Recently, it was considered that basic amino acids, including L-Arg, L-His, and L-Lys, could effectively compensate for flavor deficiencies caused by sodium reduction [7]. When 30% NaCl was replaced with KCl, alanine, citric acid, calcium lactate, and maltodextrin in reduced-sodium Harbin sausages, the undesirable metallic and harsh taste attributes associated with KCl were significantly enhanced (*p* < 0.05), while the saltness and aroma of the product were enriching [8]. Furthermore, these amino acids can not only elevate pH values, shifting them away from the pI of MPs and thereby facilitating their extraction, but they also modify the conformation of salt-soluble protein, consequently enhancing gelation properties [9,10,11]. What is more, basic amino acid bounding to acidic amino acids (Asp and Glu) on myosin may also be one of the ways that the properties of myosin are affected [12]. L-His and L-Lys made myosin unfold, causing a reduction in the α-helical structure accompanied by an increase in random coils and β-sheets, exposing hidden hydrophobic and sulfhydryl groups on the myosin surface and enhancing porcine protein solubility [13]. Jiang et al. [14] showed that the addition of only L-Arg or a combination of L-Arg and microbial transglutaminase could effectively enhance the formation of disulfide bonds and promote β-sheet structure in shrimp protein. This promotes cross-linking among the shrimp protein and enhances the overall quality of the gel. With a combination of 1% NaCl, 1.5% KCl, 1% L-Arg, and 0.2% L-His, low-sodium (40% reduction) bologna sausages have been produced with good sensory qualities and acceptability [15]. Comparing the impacts of L-Arg and L-His on the thermal gel properties of MPs, it was observed that L-Arg had a more pronounced impact than L-His [11]. It was found in the previous work that L-Arg, L-His, and L-Lys had significant effects on gel properties, and L-Arg had the best effect on improving WHC. Shi et al. [2] explored the mechanism of heat-induced myosin aggregation involving L-Arg and found that L-Arg could improve the two-phase separation caused by transitional aggregation, improve protein solubility, and ultimately improve the quality of gel products. On the whole, L-Arg gave proteins a certain shape, promoted a continuous gel network structure formation, and held the potential to replace NaCl in some low-sodium meat products.

Nevertheless, the research on the effect of partially replacing NaCl with L-Arg on the gel formation and aggregation behavior of beef myosin is quite limited. Thus, it is challenging to explore and develop research to achieve salt reduction in beef products while maintaining the gel properties of beef. This work aims to explore the influence of substituting L-Arg for NaCl on beef myosin during heating. First, we assess the impact of L-Arg on the gel properties of beef myosin from multiple aspects, such as texture, WHC, intermolecular forces, aggregation, microstructure, and rheology. Next, we measure the molecular structure changes and thermal aggregation behavior of beef myosin during heating to clarify the causes of differences in gel properties. Additionally, we investigate the underlying mechanism by which L-Arg replacing NaCl inhibits the heat-induced aggregation of myosin. This research is poised to furnish backing for the application of L-Arg in enhancing low-salt beef gels.

## 2. Materials and Methods

### 2.1. Materials

Fresh samples were acquired within 24 h after the animal’s death. The beef longissimus dorsi samples were sourced from the farmers’ market of Jiangxi Agricultural University, located in Nanchang, China. As soon as the beef is obtained, any observable fat and connective tissue are detached. The meat is then chopped to the finest state achievable. Subsequently, it is processed two times with an FSH-2A meat mincer (JingFei, Hangzhou, China) fitted possessing the perforated plate that has an inner diameter of 5 mm. Once the meat has been minced, myosin extraction is carried out right away. Throughout the entire process, the temperature is kept at 4 °C. Chemicals (Wantai Biomedicals Inc., Harbin, China) were all of no less than analytical grade quality.

### 2.2. Myosin Preparation

In accordance with the method proposed by Yu et al. [5], the extraction of beef myosin and the measurement of its concentration were carried out. The entire process was maintained at a temperature of 4 °C. Initially, 100 g of minced meat were blended in 5 times the volume of Solution A (0.1 mol/L Tris, 20 mmol/L EDTA, pH 7.0). This blend was then centrifuged at 4000× *g* for 15 min at 4 °C by means of a Velocity 14R Pro (Techcomp Scientific Instrument Co., Ltd., Shanghai, China). Subsequently, the resultant precipitate was redispersed in 3 times the volume of Solution B (0.4 mol/L KCl, 0.1 mol/L KH_2_PO_4_/K_2_HPO_4_, 2 mmol/L Na_4_P_2_O_7_, 1 mmol/L EGTA, 1 mmol/L MgCl_2_, pH 6.5) and centrifuged once more at 4000× *g* for 15 min at 4 °C. The obtained precipitate was then dissolved in 10 times the volume of highly purified water and allowed to incubate through the night. The fluffy sediments were gathered through centrifugating at 12,000× *g* for 15 min at 4 °C and then dispersed anew with Solution C (0.6 mol/L KCl, 0.15 mol/L KH_2_PO_4_/K_2_HPO_4_, 5 mmol/L Na_4_P_2_O_7_, 5 mmol/L MgCl_2_, 1 mmol/L EGTA, 1 mmol/L ATP·Na_2_, pH 7.0). After 15 min of centrifugating at 12,000× *g* and 4 °C, (NH_4_)_2_SO_4_ was gradually added to the solution containing myosin, causing the myosin to precipitate out step by step. After extraction, the myosin sample was kept at 4 °C and was used for experimental purposes within a week.

First, the myosin concentration was adjusted to 20 mg/mL. Using 0.3 M KCl to replace NaCl, seven groups with L-Arg substituting for NaCl were set up, namely A (0.3 M NaCl), B (0.25 M NaCl + 0.05 M L-Arg), C (0.2 M NaCl + 0.1 M L-Arg), D (0.15 M NaCl + 0.15 M L-Arg), E (0.1 M NaCl + 0.2 M L-Arg), F (0.05 M NaCl + 0.25 M L-Arg), and G (0.3 M L-Arg). After a thorough mixing in gel vials, the samples were heated from 25 °C to 85 °C in a water bath and held at 85 °C for 20 min. All samples were stored at 4 °C under refrigeration for about 12 h.

Two typical gels were obtained according to gel properties. C (0.2 M NaCl + 0.1 M L-Arg) had the lowest WHC and E (0.1 M NaCl + 0.2 M L-Arg) had the best microstructure. Four experimental groups, namely T1 (0.3 M NaCl), T2 (0.2 M NaCl + 0.1 M L-Arg), T3 (0.1 M NaCl + 0.2 M L-Arg), and T4 (0.3 M L-Arg), were established. The aim was to elucidate the formation of distinct gels in terms of their structural characteristics and aggregation behavior.

### 2.3. Gel Properties

#### 2.3.1. Texture Profile Analysis (TPA)

The samples were trimmed to cubes with dimensions of 25 mm in each side, making sure that all the surfaces were sleek and level. The test was carried out using a texture analyzer (Stable Micro Systems, Godalming, UK) with a P/50 probe and a cylindrical base. The test mode was set as TPA. Test parameters included pre-test, test, and post-test speeds of 1 mm/s, a compression strain of 40%, and a trigger force of 5 g. Six repetitions were carried out for each analysis.

#### 2.3.2. WHC

Around 3.0 ± 0.5 g of the gel sample (weighed as M1) was wrapped in triple layers of filter and centrifuged at 5000× *g* for 15 min at 4 °C. After centrifugation, the sample weight was recorded as M2. The WHC was calculated as follows:

(1) $$\mathrm{WHC}\;\left(\%\right)=\left[\frac{M1-M2}{M1}\right]\times 100\%$$

#### 2.3.3. Molecular Driving Forces

The molecular driving forces were gauged according to the method of Wang et al. [16], with some modification. For each sample, 2 g was taken and mixed separately with 10 mL of five solutions: S1 (0.05 mol/L NaCl), S2 (0.6 mol/L NaCl), S3 (0.6 mol/L NaCl + 1.5 mol/L urea), S4 (0.6 mol/L NaCl + 8 mol/L urea), and S5 (0.6 mol/L NaCl + 8 mol/L urea + 0.5 mol/L 2-β-mercaptoethanol). The mixtures were homogenized for 2 min, then left for 1 h to dissolve. Next, the samples were centrifuged at 10,000× *g* for 15 min at 4 °C to obtain the supernatant. For gel samples, the amounts of ionic bonds, hydrogen bonds, hydrophobic interactions, and disulfide bonds were calculated and analyzed separately, based on protein solubility differences between S2 and S1, S3 and S2, S4 and S3, and S5 and S4.

#### 2.3.4. Gel Microstructure

The myosin gel samples were cut into pieces (2 mm × 2 mm × 2 mm) and then fixed with a 2.5% (*v*/*v*) glutaraldehyde solution for 24 h. Subsequently, they were rinsed with a phosphate buffer solution (PBS, 0.2 mol/L, pH 6.0) for 15 min, and this rinsing step was repeated three times. After that, the samples were rinsed with deionized water for 1 h. Subsequently, a graded dehydration process was carried out using ethanol solutions of 50%, 70%, and 90% for 15 min each, followed by dehydration with anhydrous ethanol for 10 min, which was also repeated three times. After vacuum freeze-drying (Scientz-18NA, Ningbo Xinzhi Biotechnology Co., Ltd., Ningbo, China), the samples were sputter-coated with gold. An FEI Quanta 450 (Hillsboro, OR, USA) was employed to examined the microstructure of the myosin gel. The observation was conducted at a magnification of 5.0 K under a voltage of 15.0 kV.

### 2.4. Structure Properties

#### 2.4.1. Surface Hydrophobicity

Approximately 1 mL of the treated myosin solution with a concentration of 1 mg/mL was shaken and thoroughly blended with 200 µL of bromophenol (BPB) solution at a concentration of 1 mg/mL. As a control, 200 µL of PBS (0.2 mol/L, pH 6.0) containing bromophenol blue was added separately. The sample was centrifuged at 9000× *g* for 10 min at 4 °C in a Dynamic Velocity 14R Pro (Tech-comp Scientific Instrument Co., Ltd., Shanghai, China). Then, the obtained supernatant was diluted 10 folds with deionized water. Finally, the absorbance value was measured at a wavelength of 595 nm using a spectropolarimeter (Spectra Max M2, Molecular Devices LLC, Sunnyvale, CA, USA) to evaluate the molecular structure changes in myosin during thermal processing. The surface hydrophobicity was calculated as follows:

(2) $$\mathrm{BPB}\;\mathrm{Bound}\;\left(µg\right){=200\times (A}_{\mathrm{blank}}-A_{\mathrm{sample}}{)/A}_{\mathrm{blank}}$$

A represents the absorbance value measured at 595 nm.

#### 2.4.2. Active Sulfhydryl Groups (-SH)

The myosin solution was diluted to 0.5 mg/mL by mixing it with PBS (0.2 mol/L, pH 6.0). Then, 0.5 mL of DTNB (10 mmol/L) was added to the solution, and the mixture was allowed to react at 4 °C for 30 min. After that, the absorbance of the solution was measured at 412 nm. The content of -SH was calculated according to the following formula:

(3) $$\text{-}SH\left(µ\mathrm{mol}/100\;\mathrm{mg}\right)=\frac{A_{412}\times D\times 10^{5}}{\varepsilon \times C_{pro}}$$

In the formula, A412 is the absorbance value measured at 412 nm, D represents the dilution factor, ε refers to the molar extinction coefficient, which is 13,600 mol/L·cm, and Cpro stands for the protein concentration.

#### 2.4.3. Secondary Structure

A circular dichroism (CD) spectropolarimeter, Moss 500 (Bio-Logic, Claix, France), was used to indicate the secondary structure alteration of gels. The myosin solution, at a concentration of 0.2 mg/mL in 0.2 mol/L PBS with a pH of 6.0, was first heated up to 85 °C. The samples were allowed to equilibrate at room temperature. A quartz sample cell with an optical path length 0.1 cm was utilized. The spectral variations ranging from 190 to 260 nm were examined at the velocity of 50 nm per min.

#### 2.4.4. Tertiary Structure

PBS (0.2 mol/L, pH 6.0) was used to dilute the samples to a concentration of 0.1 mg/mL. The spectrofluorometer (F-4700, Hitachi, Japan) was employed to measure the fluorescence profiles, and fluorescence intensity (FI) readings were determined as the highest points within these profiles. The scanning parameters were arranged as follows: the excitation wavelength was set at 265 nm, the emission wavelength was scanned from 290 to 460 nm, and both the excitation and emission slits were fixed at 10 nm.

### 2.5. Aggregation Behavior

#### 2.5.1. Turbidity

A Spectra Max M2 (Silicon Valley, CA, USA) was utilized to measure the absorbance of every 0.5 mg/mL myosin solution at a wavelength of 350 nm.

#### 2.5.2. Solubility

First, the myosin solution was diluted to 1 mg/mL with 0.2 mol/L PBS at a pH of 6.0. Then, the diluted solution was spun down at 10,000× *g* for 10 min at 4 °C. Protein solubility was calculated as the ratio of the protein amount in the centrifuged supernatant to that prior to centrifugation.

#### 2.5.3. Dynamic Light Scattering (DLS)

The DLS of gels was measured according to the method of Yan et al. [17] with minor modifications. The myosin concentration was adjusted to 0.1 mg/mL with 0.2 mol/L PBS at a pH of 6.0. Each sample was then heated from 25 °C to 85 °C at a rate of 1 °C/min, and then 25 °C, 45 °C, and 75 °C were selected to compare the particle size differences in the samples. During the programmed temperature increase, monitoring was carried out using the Cumulants model.

#### 2.5.4. Aggregation Characteristics

The aggregation characteristics of gels were measured according to the method of Gao et al. [11] with minor modifications. The myosin concentration was adjusted to 0.05 µg/mL with PBS (0.2 mol/L, pH 6.0). An appropriate sample solution was absorbed on the mica sheet surface and dried at 25 °C. The AFM instrument was BRUKER ICON with probe model OTESPA-R3. The test mode was tapping in air, and the resolution was 256 × 256. The sample map scanning range was 2 µm × 2 µm and could be narrowed as per the actual situation for a more accurate depiction of sample changes. Then, 25 °C, 45 °C, and 75 °C were selected to compare the aggregation characteristic differences of the samples. Software NanoScope Analysis 3.00 was used to process the sample images, with only the flatten function employed to obtain 2D and 3D images.

### 2.6. Statistic Analysis

The experiment was repeated thrice, the data used SPSS 26.0, and Duncan method was used for multiple comparisons, with *p* < 0.05 indicating a significant difference. Moreover, data were expressed as means ± standard deviations. All of the graphs presented were plotted using Origin 2018b.

## 3. Results and Discussion

### 3.1. Gel Properties

#### 3.1.1. TPA

The overall quality of processed meat products is significantly influenced by their texture properties, which serve as key indicators for assessing the product’s quality.

As shown in Table 1, the substitution of NaCl with L-Arg led to significant enhancements (*p* < 0.05) of hardness, springiness, cohesiveness, and gumminess. Group E exhibited the values for hardness (214.274 ± 11.519 g) and springiness (1.336 ± 0.003), respectively. These two values are not significantly different from the values in groups F and G. These values had no significant difference when the substitution levels continued to increase beyond the level in group E. Meanwhile, the cohesiveness and gumminess both reached their maximum values (0.762 ± 0.05 and 185.014 ± 7.118 g) in Group E, and then both declined with further higher L-Arg substitution. It could be because the positive charge on the L-Arg side chain partly took over the ionic action of NaCl, facilitating the formation of salt bridges that enhanced the gel TPA [18]. However, excess L-Arg substitutions do not have an enhanced effect on TPA, resulting from the charge and steric hindrance effects inside the gel [19]. Overall, replacing NaCl with L-Arg in meat processing might enhance meat texture, particularly hardness and springiness. This may be achieved by altering beef myosin conformation and intermolecular forces, while keeping cohesiveness and gumminess at acceptable levels.

#### 3.1.2. WHC

**Figure 1:**
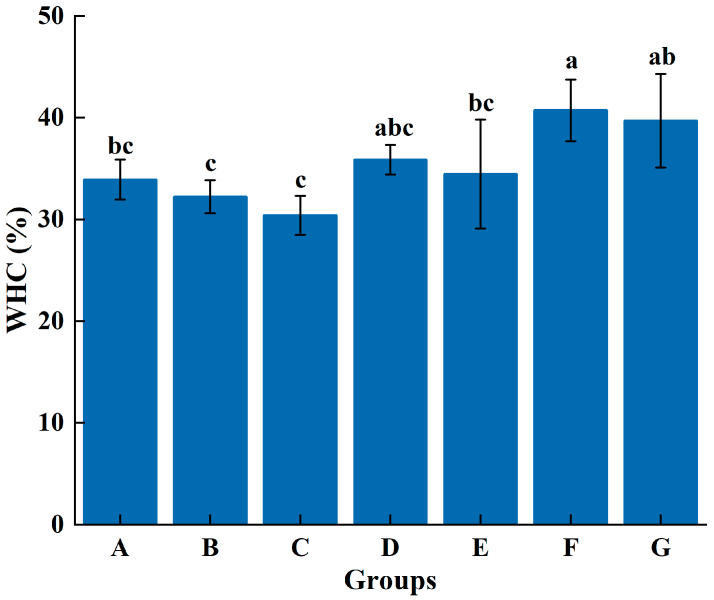
Effect of L-Arg substitution of NaCl on WHC of myosin gel. Note: A (0.3 M NaCl), B (0.25 M NaCl + 0.05 M L-Arg), C (0.2 M NaCl + 0.1 M L-Arg), D (0.15 M NaCl + 0.15 M L-Arg), E (0.1 M NaCl + 0.2 M L-Arg), F (0.05 M NaCl + 0.25 M L-Arg), and G (0.3 M L-Arg). a–c indicates a significant difference (*p* < 0.05) among samples of A–G groups.

As can be seen from Figure 1, the substitution of NaCl with L-Arg in myosin gels resulted in notable enhancements in WHC. And a marked positive correlation was observed between L-Arg concentration and WHC (*p* < 0.05). This phenomenon might be due to the fact that the positive charge carried by L-Arg might interact with negative charges in myosin, promoting cross-linking of protein molecules and then assisting in forming stable and robust gel network conformation [18,20]. These interactions aided in trapping water molecules within the gel matrix, enhancing the WHC of beef myosin gel [21]. Interestingly, despite the highest moisture content in groups F and G, their gumminess was lower than that of group E (34.5 ± 5.37%). This indicated that while water retention was important, it was not the sole determinant of gel quality. The gel quality of group E, which exhibited a balance between hardness, elasticity, and WHC, was found to be the best among the tested groups.

#### 3.1.3. Molecular Driving Forces

**Figure 2:**
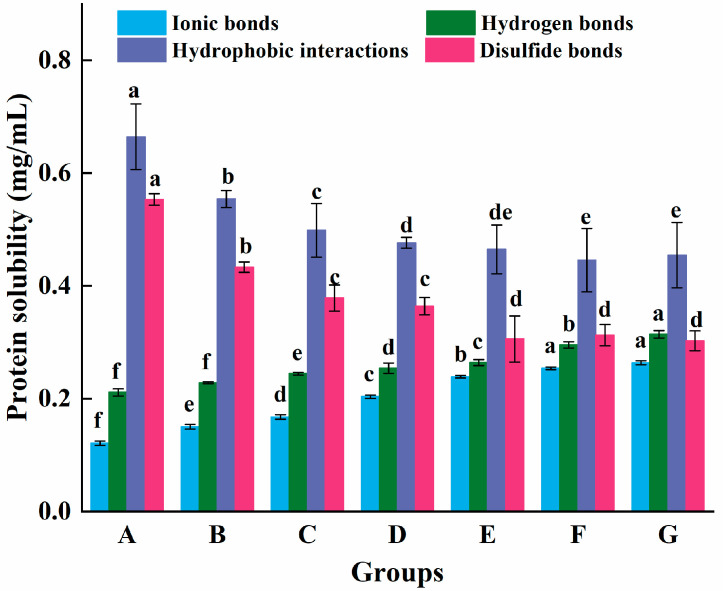
Effect of the L-Arg substitution of NaCl on the soluble protein content of myosin gel. Note: A (0.3 M NaCl), B (0.25 M NaCl + 0.05 M L-Arg), C (0.2 M NaCl + 0.1 M L-Arg), D (0.15 M NaCl + 0.15 M L-Arg), E (0.1 M NaCl + 0.2 M L-Arg), F (0.05 M NaCl + 0.25 M L-Arg), and G (0.3 M L-Arg). a–f indicates a significant difference (*p* < 0.05) among samples of A–G groups.

In Figure 2, hydrogen and ionic bond contents were significantly lower compared to the hydrophobic interaction and disulfide bond contents in all samples. This finding meant that hydrophobic interactions and disulfide bonds assume a preponderant role in maintaining stable beef myosin gels. It could be that myosin was denatured when heating, thus unfolding the structure and exposing the hydrophobic and sulfhydryl groups, which facilitated protein cross-linking. It was noted that the results were comparable to the changes in gel qualities. Jiang et al. [14] also found that adding L-Arg was able to enhance the surface hydrophobicity in reduced-salt white *Litopenaeus vannamei* shrimps. Ye, Zhang, Deng, Li, and Chen [22] suggested that adding L-Arg could increase disulfide bonds because the double bond structure on the L-Arg guanidine group may strip hydrogen atoms from the sulfhydryl group. In this study, the decrease in disulfide bond content was hypothesized to result from L-Arg binding to myosin, which increased the distance between Cys residues for disulfide bond formation. This hypothesis is based on two speculations. First, the steric hindrance of L-Arg, upon binding, may push apart adjacent Cys due to its size and shape. Second, L-Arg binding can induce myosin conformational changes, distorting the protein structure and increasing the distance between Cys residues primed for disulfide bond formation. Furthermore, it was determined that the stable conformation of myosin gels was not primarily driven by hydrogen bonding or ionic bonds. Nevertheless, their role significance grew as the percentage of L-Arg substitution rose. The system ionic strength and charge distribution might change when L-Arg partly substituted NaCl, and the hydroxyl and guanidine groups of L-Arg could better establish hydrogen bonds [23].

#### 3.1.4. Gel Microstructure

**Figure 3:**
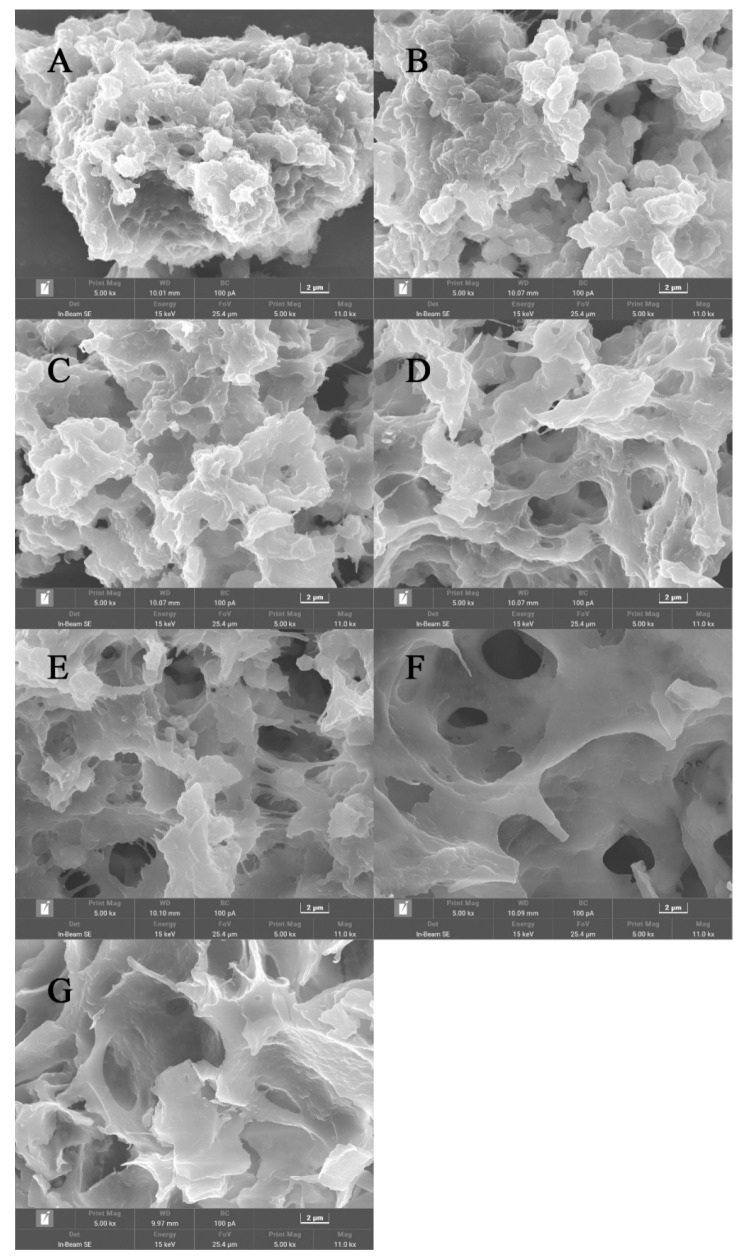
Effect of the L-Arg substitution of NaCl on the gel microstructure of myosin gel (magnification, 5000×). Note: (**A**) (0.3 M NaCl), (**B**) (0.25 M NaCl + 0.05 M L-Arg), (**C**) (0.2 M NaCl + 0.1 M L-Arg), (**D**) (0.15 M NaCl + 0.15 M L-Arg), (**E**) (0.1 M NaCl + 0.2 M L-Arg), (**F**) (0.05 M NaCl + 0.25 M L-Arg), and (**G**) (0.3 M L-Arg).

As demonstrated in Figure 3, the L-Arg substitutions resulted in notable alterations in beef myosin gel networks observed by SEM, presumably due to the role of L-Arg in modifying beef myosin gel microstructure and properties.

The gel network with NaCl alone exhibited a coarse layered structure. Noticeably, upon adding L-Arg, the beef myosin gel network structure transformed into a porous structure. This transformation became more pronounced with increasing L-Arg substitution, indicating a positive correlation between the substitution and the porosity of the structure Gao et al. [24]. Notably, in group E, the layered structure within the gel network was significantly reduced compared to group A. L-Arg was able to form hydrogen bonds with beef myosin, thus locking more water into the beef myosin gel network, causing a porous and elastic gel [25]. Furthermore, L-Arg might have facilitated stronger interactions between myosin molecules, inducing a more uniform gel [11]. However, in groups F and G, fragmentation of the gel structure was observed. This was likely due to excessive L-Arg substitution, resulting in overly strong interactions between L-Arg and beef myosin; interactions might destroy connections among myosin molecules. Above all, the microstructure of group E was greater in all treatment groups.

### 3.2. Structure Properties

#### 3.2.1. Surface Hydrophobicity

**Figure 4:**
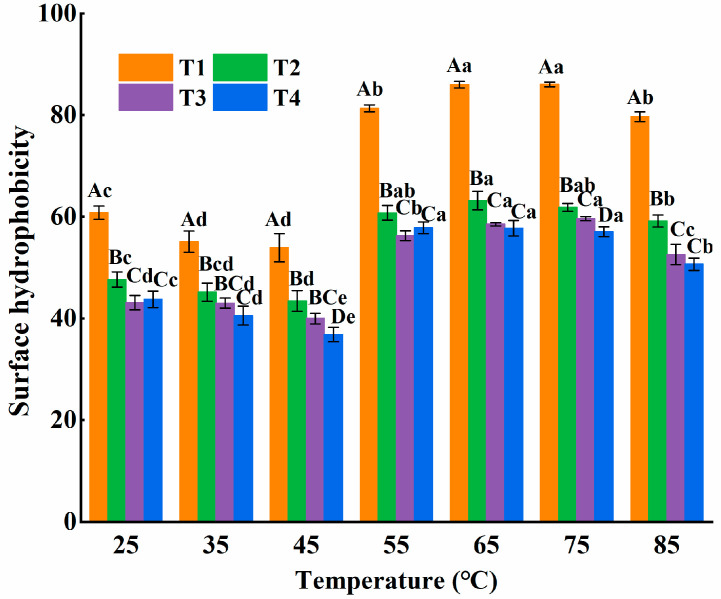
Effect of L-Arg substitution of NaCl on the surface hydrophobicity of myosin. Note: T1 (0.3 M KCl + 0.3 M NaCl), T2 (0.3 M KCl + 0.2 M NaCl + 0.1 M L-Arg), T3 (0.3 M KCl + 0.1 M NaCl + 0.2 M L-Arg), and T4 (0.3 M L-Arg). Results are presented as mean ± standard deviation (M ± SD) (n = 3). Capital letters indicate significant differences among different groups under the same temperature condition (*p* < 0.05), while lowercase letters denote significant differences between different temperatures within the same group (*p* < 0.05).

Surface hydrophobicity serves as a crucial metric for demonstrating the arrangement of hydrophobic groups that are exposed on the surface of the protein. As the temperature increased, the surface hydrophobicity of all four groups generally showed a pattern of initially decreasing and then rising in Figure 4. At the early stage of heating, the structure of myosin began to unfold. L-Arg interacted with myosin via covalent and non-covalent bonds to form reversible aggregates, which might partially cover some hydrophobic groups. As the temperature continued to rise, the stability of the aggregates decreased, thus exposing hydrophobic groups. It was notable that when the temperature reached 65 °C, the surface hydrophobicity of myosin in each of the four groups attained its peak values. Precisely, for group T1, the value was 85.97 ± 0.65; for group T2, it was 63.17 ± 1.84; for group T3, it was 58.5 ± 0.33; and for group T4, it was 57.75 ± 1.53. When the temperature reached about 85 °C, the significant decrease (*p* < 0.05) was observed in the surface hydrophobicity of myosin across all four groups. The increase in protein–protein interactions led to the high-molecular-weight aggregates, partially masking the unfolding effect, thereby reducing the hydrophobicity of the protein surface [26].

In Figure 4, over the entire course of heating, the surface hydrophobicity of myosin in the non-substituted group was notably higher than that in the L-Arg substituted group (*p* < 0.05). Due to its low ionization degree, the ionic strength in the microenvironment was reduced, disturbing the full expansion of the protein structure. Thus, the content of exposed hydrophobic groups exhibited a marked reduction in comparison to the non-substituted group (*p* < 0.05). Meanwhile, the surface hydrophobicity decreased with the increase in L-Arg in replacing NaCl. Gao, Wang, Mu, Shi and Yuan [27] found that L-his weakened the hydrophobic interaction by affecting the charge distribution of the amino acid side chain of myosin and inhibited the aggregation among myosin, thus contributing to the formation of finer protein aggregates and honeycomb three-dimensional structures. It could be speculated that L-Arg might have an impact on the exposure of carbonyl and amide groups on the carbon skeleton of myosin. That is, L-Arg might react with these groups or amino acids nearby [28]. Additionally, the guanidine group on the R-group of L-Arg might replace water molecules, forming a protective shield around the exposed hydrophobic residue, thereby reducing the hydrophobic interaction required for myosin thermal aggregation [29].

#### 3.2.2. -SH

**Figure 5:**
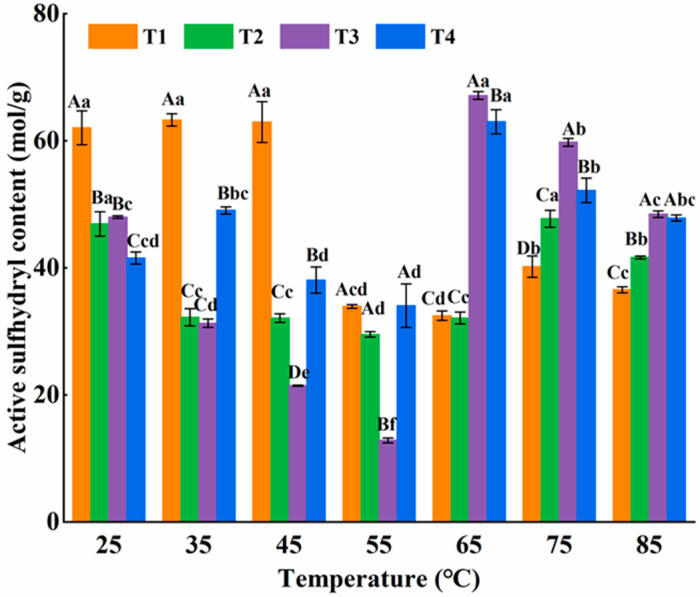
Effect of L-Arg substitution of NaCl on active sulfhydryl content of myosin. Note: T1 (0.3 M KCl + 0.3 M NaCl), T2 (0.3 M KCl + 0.2 M NaCl + 0.1 M L-Arg), T3 (0.3 M KCl + 0.1 M NaCl + 0.2 M L-Arg), and T4 (0.3 M L-Arg). Results are presented as mean ± standard deviation (M ± SD) (n = 3). Capital letters indicate significant differences among different groups under the same temperature condition (*p* < 0.05), while lowercase letters denote significant differences between different temperatures within the same group (*p* < 0.05).

The content of active sulfhydryl groups is regarded as a crucial index for uncovering the generation of disulfide bonds. As depicted in Figure 5, with the temperature increasing, the quantity of active sulfhydryl groups in the four groups of samples initially showed a downward trend, then went up, and eventually dropped slightly. Du et al. [26] conducted research on the sulfhydryl group content in the myofibrillar of mirror carp. It was found that when the samples underwent heat treatment at temperatures higher than 40 °C, a decrease in the sulfhydryl group content ensued. When the temperature was below 55 °C, the sulfhydryl groups exposed by protein folding oxidized to build disulfide bonds, resulting in a reduction in sulfhydryl content. When above 55 °C, the content of active sulfhydryl groups in four groups began to increase. Myosin tail denaturation potentially disrupts the pre-formed reversible and unstable gel among myosin heads. This conformational alteration of the myosin molecule leads to the exposure of formerly covering sulfhydryl groups, consequently augmenting the quantity of active sulfhydryl moieties [5]. When at 85 °C, the high temperature could cause changes in the interactions between myosin molecules and between L-Arg and protein molecules, destroying the already formed gel structure and leading to a reduction in disulfide bonds [26].

When the temperature was above 55 °C, the amount of active sulfhydryl group in the L-Arg substitutions was higher than that in the group without L-Arg. As reported by Lei et al. [21], L-Arg could enhance the number of surface sulfhydryl groups and the water retention of chicken actomyosin. It is postulated that the amino NH_2_ and other functional groups of L-Arg initially interact with-OH, thereby inhibiting L-Arg-induced myosin molecule unfolding. This inhibits the exposure of the buried sulfhydryl groups on the protein surface, reducing their susceptibility to hydroxyl radical attack [2]. This most likely decreased the contribution of the disulfide bond in the myosin gel. This phenomenon was most prominent in T3 (0.3 M KCl + 0.1 M NaCl + 0.2 M L-Arg) (*p* < 0.05). At 65 °C, the active sulfhydryl group’s content of myosin in T3 reached 67.11 ± 0.59 mol/g.

#### 3.2.3. Secondary Structure

**Figure 6:**
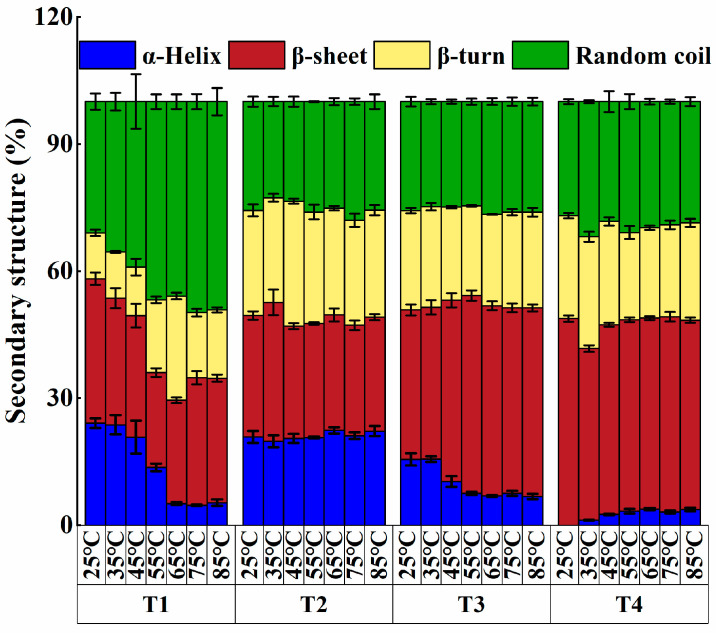
Effect of L-Arg substitution of NaCl on the secondary structure of myosin. Note: T1 (0.3 M KCl + 0.3 M NaCl), T2 (0.3 M KCl + 0.2 M NaCl + 0.1 M L-Arg), T3 (0.3 M KCl + 0.1 M NaCl + 0.2 M L-Arg), and T4 (0.3 M L-Arg).

As shown in Figure 6, the protein secondary structure mainly consists of three regular structures: the α-helix, β-sheet, and β-turn, while the rest are random coils.

In the T1 group, following the temperature upsurge, the α-helix structure content in myosin molecules gradually decreased, while the content of β-sheet, β-turn, and random coil structures increased. This phenomenon might be attributed to the combined effect of salt ions and heating on protein molecules, which alters protein internal interaction forces and protein stability, leading to the rearrangement of secondary structures. Notably, a similar tendency was observed in the L-Arg substitution group. Compared with the control group, the myosin of L-Arg substitutions was more likely to shift from the α-helix structure to the β-sheet and β-turn structures, and the amount of random coil was notably reduced (*p* < 0.05). In the T2 group, the random coil content of myosin decreased significantly at 25 °C (*p* < 0.05) compared with the T1 group, and the content of the α-helix structure increased markedly throughout the heating process (*p* < 0.05). It was speculated that L-Arg can prompt the transformation of α-helix to other secondary structures during heating. Additionally, it may lead to refolding or rearrangement of some non-helical regions of the protein during the heating process, reforming a regular secondary structure within the protein (transforming some random coil to α-helix, β-sheet, and β-turn). This may be because, as an alkaline amino acid, L-Arg has a positive charge on its side chain that can interact with the negative charge in the protein molecule, thereby influencing the electric repulsion among proteins as well as the formation of hydrogen bonds [30]. Thus, those interactions might prompt the transformation of the irregular curled region to a more orderly α-helix, β-sheet, and β-turn structure. In group T3, as the L-Arg substitution ratio rose, the ionic strength of the myosin system further declined, the α-helix structure content reduced, and the β-sheet structure content further augmented, which was important for protein aggregation [31]. At 55 °C, the β-sheet structure content of myosin attained the maximum value (46.67 ± 1.22%). At 45 °C, the α-helix structure of T3 was mainly converted into the β-sheet structure, while at 65 °C, the α-helix structure was mainly transformed into the β-sheet and random coil structures. Thus, myosin of the T3 group mainly occurred in the conversion of the regular secondary structure; however, it would be accompanied by an increase in the amount of the irregular curled structure at high temperatures. In the T4 group, the α-helix and β-sheet structures of myosin were mainly converted into random coiled structures about 45 °C and 65 °C. This is in line with the finding of Shi et al. [2], who found that L-Arg promoted the transformation of the regular secondary structure to the random coil of myosin during heating. L-Arg could promote the interconversion of regular and irregular secondary structures in myosin. Therefore, L-Arg could open protein molecules, facilitating the deconstruction of beef myosin molecules. Meanwhile, it effectively augmented the β-sheet content and reduced the random coil content, which might be the reason for its ability to inhibit the cross-linking of beef myosin molecules.

#### 3.2.4. Tertiary Structure

Maximum fluorescence emission wavelength (λmax) and FI are often used to characterize the changes in protein microenvironment and the polarity of tryptophan (Trp) [32]. The alterations within the tertiary conformation of myosin during the heating process after L-Arg were further substituted for NaCl, as shown in Table 2.

The λmax of all samples was greater than the maximum λmax (330 nm) of Trp, which indicated that the tertiary structure of myosin in all groups changed. Specifically, during the whole heating process, myosin partially or completely unfolded, resulting in changes in internal structure. Trp residues were more exposed to the surface of protein molecules in a hydrophilic environment, resulting in a decrease in the FI of excited Trp and a redshift in the maximum wavelength [33]. It can be seen from the data in Table 2 that the addition of L-Arg may affect the tertiary structure of myosin with the assistance of temperature.

It could be observed that after L-Arg was added to replace NaCl, the FImax of myosin during the heating process underwent significant changes, indicating that L-Arg could bind to myosin and alter the protein structure. According to Table 2, the FI of myosin in the T1 group increased significantly with the rise in temperature (*p* < 0.05). When heated, myosin began to unfold, and exposing the buried Trp to a hydrophilic environment caused a low FI in the T1 group at lower temperatures. At 45–55 °C, a head-to-head cross-linking reaction among myosin caused chromophores to re-embed into protein molecules, preventing their exposure and increasing FI. When the temperature was 65–75 °C, the FI of myosin decreased to 361.9 ± 4.7. As the temperature further increased, although tail changes in myosin disrupted the reversible aggregation between some head parts, stable cross-linking was dynamically and simultaneously formed between the tail and the head-tail, resulting in a relatively stable gel and preventing FI quenching. Therefore, the FI remained at a high state. It was noteworthy that when L-Arg replaced NaCl, the trend of FI change with temperature in the substitution groups was similar to that in the T1 group, yet there were slight differences. The FI of the T2 and T3 groups at high temperature was higher than that at low temperature. This indicated that the myosin structure was relatively loose at low temperatures, while the gel was relatively compact at high temperatures, wrapping the chromophore group inside. However, the FI in the T4 group at high temperature was lower than that at low temperature. It might be that after L-Arg completely replaced NaCl, owing to the hydrophobic interaction and the decrease in disulfide bonds, the proportion of hydrogen bonds and ionic bonds within the formed gel was enhanced. Nevertheless, it was readily damaged by high temperatures, exposing Trp to the hydrophilic environment and causing a certain degree of FI quenching [26].

### 3.3. Aggregation Behavior

#### 3.3.1. Turbidity

**Figure 7:**
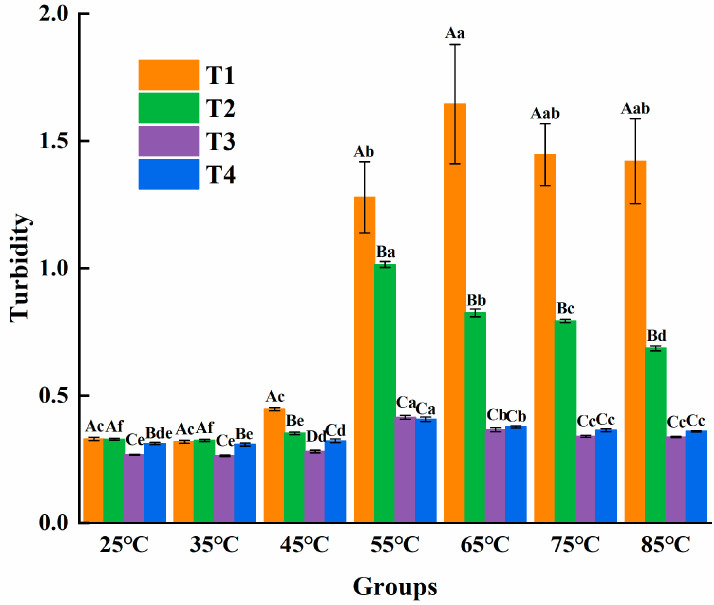
Effect of L-Arg substitution of NaCl on turbidity of myosin. Note: T1 (0.3 M KCl + 0.3 M NaCl), T2 (0.3 M KCl + 0.2 M NaCl + 0.1 M L-Arg), T3 (0.3 M KCl + 0.1 M NaCl + 0.2 M L-Arg), and T4 (0.3 M L-Arg). Results are presented as mean ± standard deviation (M ± SD) (n = 3). Capital letters indicate significant differences among different groups under the same temperature condition (*p* < 0.05), while lowercase letters denote significant differences between different temperatures within the same group (*p* < 0.05).

In general, the turbidity values of myosin in the four groups demonstrated a trend of first rising and then falling in Figure 7. All four treatment groups began to increase markedly at 55 °C. Among them, the turbidity value of T1 attained the maximum at 65 °C (1.64 ± 0.23), which demonstrated a remarkably greater value compared to what the other groups had (*p* < 0.05). In the T1 group, the reason why the turbidity is relatively high there is probably because myosin tends to clump together on its own when there is a certain amount of NaCl around. When the thermal denaturation temperature was reached, myosin molecules unfolded, and then intermolecular aggregation took place under driving forces like hydrophobic interaction and disulfide bond. As the temperature increased further, the turbidity of each group would decline. It might be that rapid and intense aggregation caused myosin particles to settle, enabling light to penetrate the supernatant [34]. When using L-Arg to replace NaCl, L-Arg interacts with myosin in a way that seems to stop it from clumping so much that the turbidity of the L-Arg–myosin solution decreases. Once we replace NaCl with L-Arg beyond a certain amount, the turbidity does not change much. This shows that the interaction between L-Arg and myosin has reached a kind of balance. L-Arg changed the conformational of myosin tail, resulting in a periodic charge imbalance that inhibited the excessive aggregation of filaments.

During the heating process, the turbidity of myosin in the T1 group consistently remained greater than that in the substitution group. Furthermore, the turbidity of the L-Arg-myosin solution decreased as the L-Arg replacement ratio increased; the turbidity levels of myosin in both the T3 and T4 groups were constantly less than those in the other groups. It is noteworthy that when L-Arg replaced NaCl beyond a certain proportion, the turbidity did not change significantly. The addition of basic amino acids could weaken the aggregation of myofibrillar protein and lead to a reduction in turbidity [35]. Hence, it was speculated that L-Arg and myosin could interact to lower the turbidity in solution.

#### 3.3.2. Solubility

**Figure 8:**
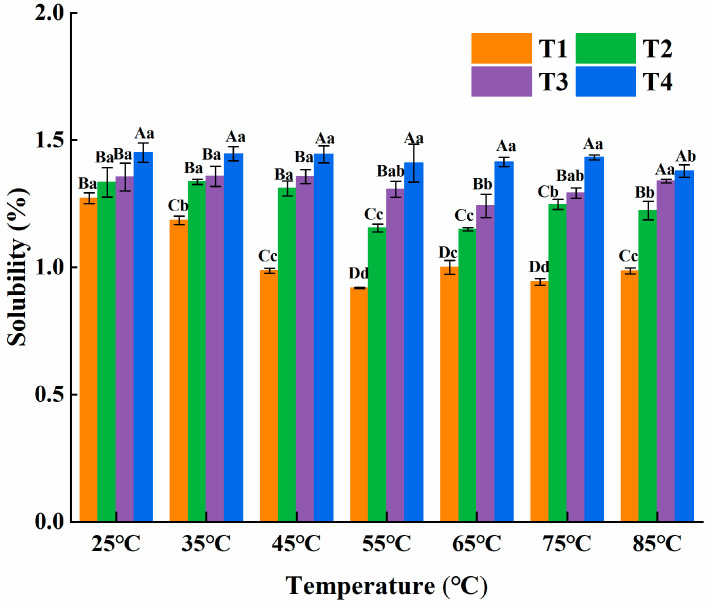
Effect of L-Arg substitution of NaCl on solubility of myosin. Note: T1 (0.3 M KCl + 0.3 M NaCl), T2 (0.3 M KCl + 0.2 M NaCl + 0.1 M L-Arg), T3 (0.3 M KCl + 0.1 M NaCl + 0.2 M L-Arg), and T4 (0.3 M L-Arg). Results are presented as mean ± standard deviation (M ± SD) (n = 3). Capital letters indicate significant differences among different groups under the same temperature condition (*p* < 0.05), while lowercase letters denote significant differences between different temperatures within the same group (*p* < 0.05).

As the temperature rose, myosin solubility showed a downward tendency in Figure 8. During the heating process, myosin underwent denaturation and aggregation, resulting in a reduction in its solubility. Additionally, the higher the substitution ratio of L-Arg, the greater the solubility of the myosin system. Under the experimental conditions, myosin mostly existed as myosin monomers and conversed to myosin filaments. L-Arg could hardly inhibit the formation of filaments, but it could bind to myosin monomers and enhance the equilibrium solubility of myosin monomers. Takai, Yoshizawa, Ejima, Arakawa, and Shiraki [36] believed that the combination of L-Arg and myosin monomer improves the activation energy of myosin monomer to form filament polymer. Thus, it could be concluded that L-Arg enhanced the solubility of myosin under certain conditions.

#### 3.3.3. DLS

**Figure 9:**
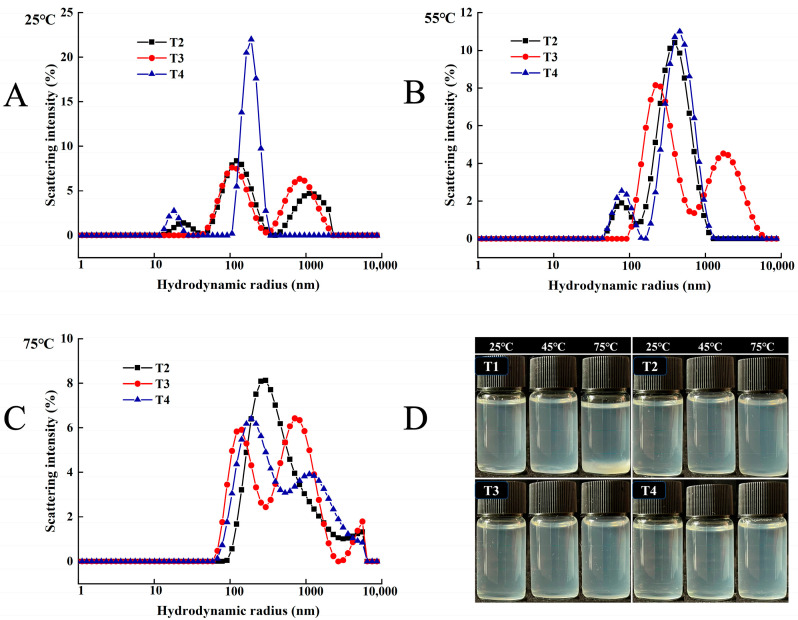
Effect of L-Arg substitution of NaCl on particle size of myosin. Note: T1 (0.3 M KCl + 0.3 M NaCl), T2 (0.3 M KCl + 0.2 M NaCl + 0.1 M L-Arg), T3 (0.3 M KCl + 0.1 M NaCl + 0.2 M L-Arg), and T4 (0.3 M L-Arg). (**A**–**C**) The change in the particle size of myosin in Groups T2–T4 under the conditions of 25 °C, 55 °C, and 75 °C, respectively. (**D**) Visual diagrams of Groups T1–T4 under the conditions of 25 °C, 55 °C, and 75 °C, respectively.

In the heating process of biopolymers/mixtures, phase separation frequently occurs due to intermolecular interactions. As shown in Figure 9D, the myosin in the T1 group formed visible precipitation, indicating that the protein in the control group was heat-denatured with its structure unfolding and then precipitation or aggregation occurring. Therefore, the aggregation status of myosin in the TI group could not be obtained by DLS. The phase separation was improved with the addition of L-Arg, and it has been reported that using L-Arg as an additive could dissolve insoluble myosin in physiological saline solution [36].

As shown in Figure 9A–C, myosin in the three substitution groups coexists in different forms. At 25 °C, the position of the myosin peak decreased to less than 1000 nm with protein deconstruction and maintenance at the filament level. The higher the concentration, the more pronounced the effect. Myosin exists as monomers, a large number of soluble filaments, and complexes between soluble oligomers and soluble filaments [37]. At 25 °C, L-Arg could induce protein deconstruction, and the union of L-Arg with myosin to form L-Arg-myosin monomer complex may also increase the particle size. Meanwhile, the presence of L-Arg can hardly inhibit the formation of filaments.

At 55 °C, the peak position in the three substitution groups shifted to the right. However, myosin was predominantly below 1000 nm. In T2 and T4, myosin formed head aggregations with the coexistence of monomers and filaments. The peak position of myosin in T3 shifted to the right, some particle sizes increased, and soluble protein oligomers were detected in T3 at 1720 nm. The results indicated that soluble aggregates dominated by filaments and oligomers were formed between the myosin heads. The particle size of myosin in substitutions exhibited a marked decrease compared to the T1 group (in Figure 9D). When the temperature reached 55 °C, protein head–head cross-linking occurred. Replacing NaCl with L-Arg enhanced the solubility of myosin and inhibited the disordered and excessive aggregation of myosin, enabling myosin to maintain the states of monomer, filament, and oligomer to participate in head aggregation.

At 75 °C, the mean particle size of myosin further decreased significantly (*p* < 0.05), speculating that replacing NaCl with L-Arg. inhibited tail aggregation to a greater extent. Changes in the tail conformation of myosin could lead to periodic charge imbalance, which inhibited the formation of filaments [38]. Above the thermal denaturation temperature, the soluble filaments dissociated and unfolded to form soluble oligomers and bound to the persistent soluble filaments. Thus, it could be inferred that the myosin thermal gel in the L-Arg-KCl-NaCl sodium reduction system was mainly composed of myosin oligomers and myosin filaments.

#### 3.3.4. Aggregation Characteristics

**Figure 10:**
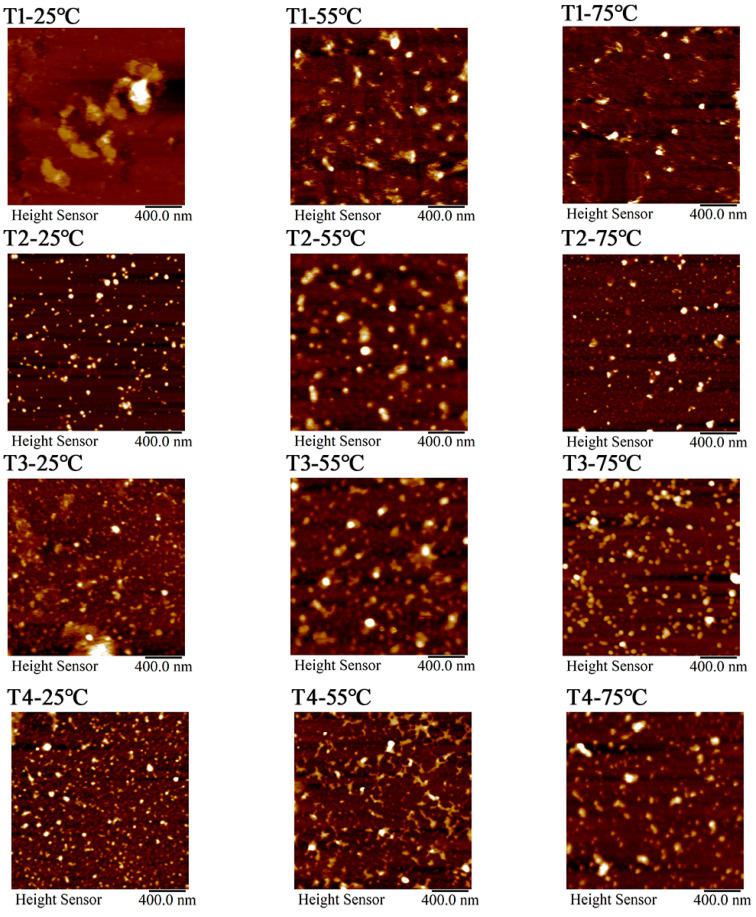
Effect of L-Arg substitution of NaCl on AFM micrographs (2D) of myosin. Note: T1 (0.3 M KCl + 0.3 M NaCl), T2 (0.3 M KCl + 0.2 M NaCl + 0.1 M L-Arg), T3 (0.3 M KCl + 0.1 M NaCl + 0.2 M L-Arg), and T4 (0.3 M L-Arg).

**Figure 11:**
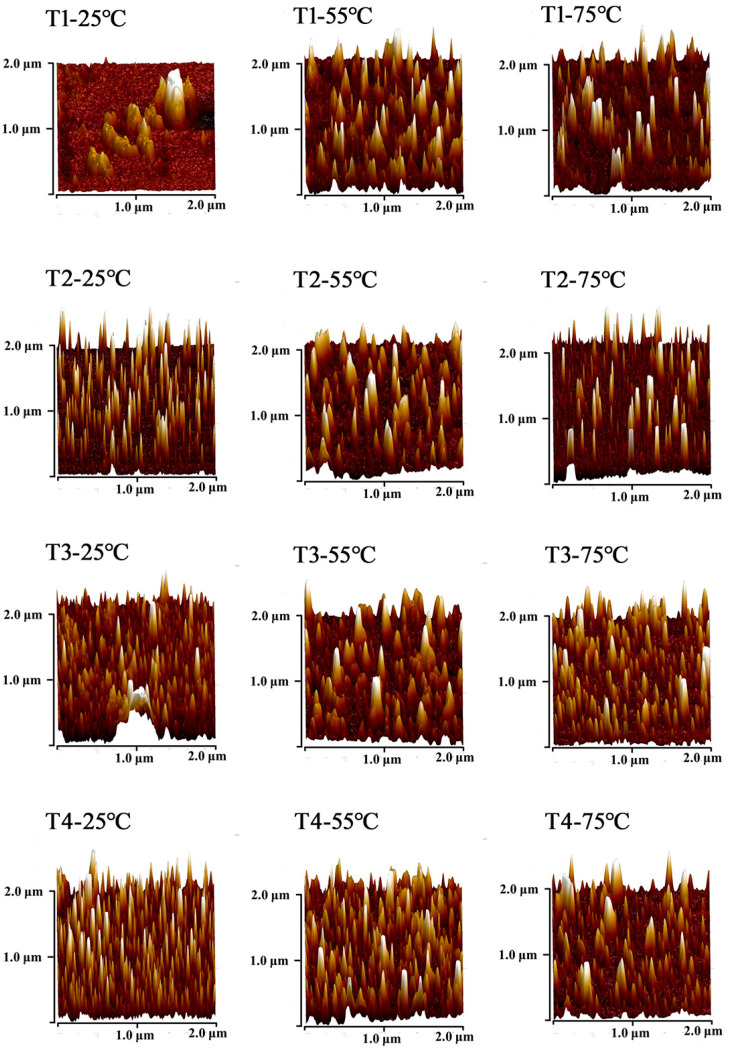
Effect of L-Arg substitution of NaCl on AFM micrographs (3D) of myosin. Note: T1 (0.3 M KCl + 0.3 M NaCl), T2 (0.3 M KCl + 0.2 M NaCl + 0.1 M L-Arg), T3 (0.3 M KCl + 0.1 M NaCl + 0.2 M L-Arg), and T4 (0.3 M L-Arg).

**Figure 12:**
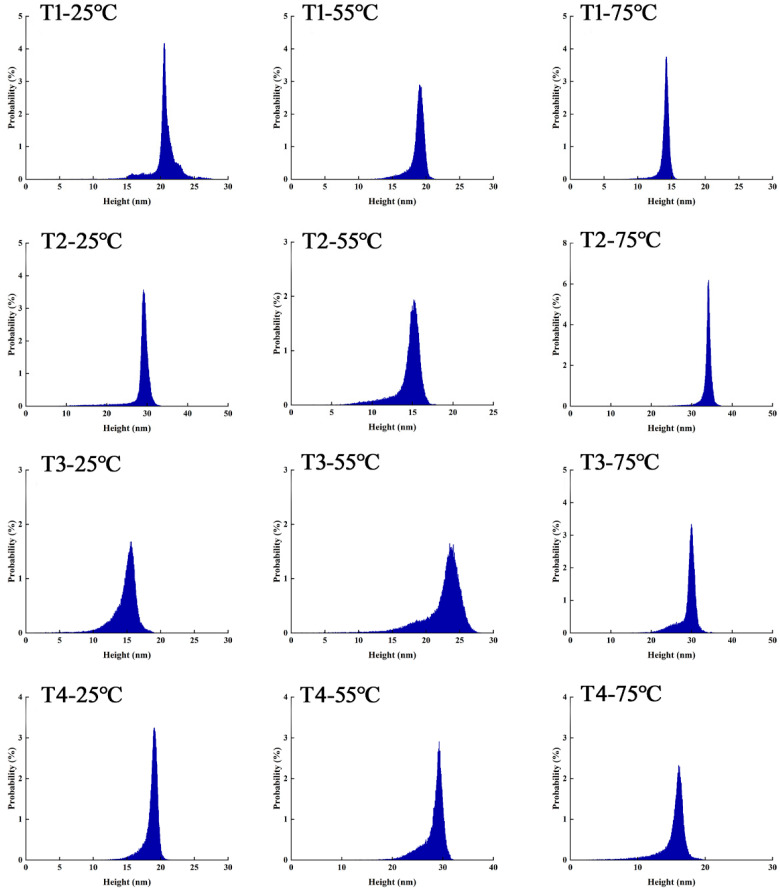
Effect of L-Arg substitution of NaCl on AFM micrographs (height distribution) of myosin. Note: T1 (0.3 M KCl + 0.3 M NaCl), T2 (0.3 M KCl + 0.2 M NaCl + 0.1 M L-Arg), T3 (0.3 M KCl + 0.1 M NaCl + 0.2 M L-Arg), and T4 (0.3 M L-Arg).

At 25 °C, myosin had a certain degree of dispersion in the 0.6 M NaCl system. Myosin predominantly exists in the forms of monomers, filaments, and some small aggregates. As shown in Figure 10, Figure 11 and Figure 12, the aggregates were highly concentrated in the range of 20–25 nm. As L-Arg further replaced NaCl, there were myosin monomers that appeared, and their number was positively correlated with the L-Arg replacement ratio.

When the temperature rose to 55 °C, myosin fully unfolded, mostly in the form of small aggregates. Moreover, the transverse cross-linking further increased among myosin.

The height of aggregates was concentrated within the range of 15–20 nm. The results demonstrated that myosin in the control group was heated, denatured, deconstructed, and aggregated. The addition of L-Arg effectively inhibited the disordered aggregation among myosin aggregates, and the transverse cross-linking of myosin was weakened. Myosin mostly exists in protein clusters formed by monomers and microaggregates. Notably, myosin microaggregates in the T3 group were evenly distributed, and the height range of the aggregates was concentrated around 15 nm. However, in the T4 group, the shape of myosin aggregates changed significantly with the appearance of linear aggregates, and there were slight cross-links among them, resulting in a network structure with relatively uniform dispersion. When the temperature rose to 75 °C, tail denaturation destroyed the reversible aggregates formed the head–head. The local small aggregates were slightly cross-linked, and some regions were compacted into separate fragments, resulting in the disappearance of the network structure and some patches. In the partial substitutions, the transverse cross-linking of myosin was weakened, and myosin mostly existed as protein clusters formed by monomers and small aggregates, which were more uniform. In the substitutions, T3-75 °C had relatively uniform and regular myosin aggregates, denoting that myosin had a tendency to develop a good gel network structure under this condition. After L-Arg further replaced NaCl, it could effectively inhibit the disordered aggregation of myosin and enable myosin to participate in the gel formation process in the form of monomers or small aggregates.

## 4. Mechanistic Explanation

**Figure 13:**
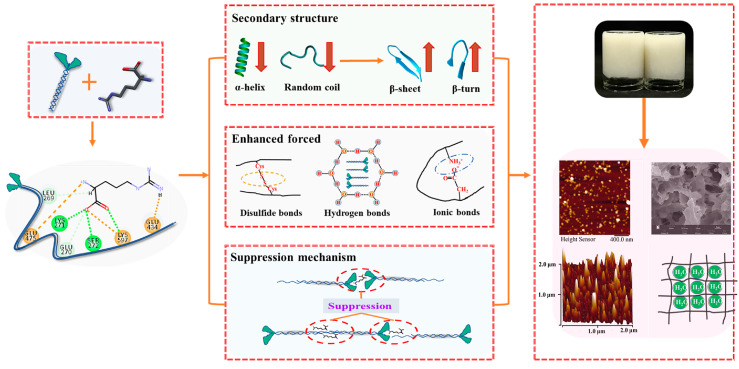
Underlying mechanism of the L-Arg substitution of NaCl on the heat aggregation behavior and gel properties of myosin.

After further substituting NaCl with L-Arg, based on the impacts of L-Arg regarding the thermal gel properties and aggregating behavior of beef myosin, a potential mechanism was shown in Figure 13. In the sodium reduction system, L-Arg is bound to myosin monomer, thereby increasing the myosin particle size and increasing the activation energy of myosin for filament polymer formation. Thus, L-Arg could enhance the solubility of myosin at a certain ion concentration. At 25 °C, L-Arg affected the structure of the myosin head, leading to its deconstruction at room temperature. At this point, the contribution of the hydrophobic interaction between proteins and disulfide bonds showed a marked decrease compared to those in the control group (*p* < 0.05), implying that L-Arg could promote head unfolding while inhibiting aggregation of the myosin head. According to the particle size results, the myosin in the group without L-Arg could not be detected by the particle size analyzer, whereas the particle size in the substitutions could be detected. When the temperature rose (55 °C), L-Arg combined with the amino acids of myosin through hydrogen bonds and electrostatic interactions, thus altering the internal structure of myosin, reducing the content of α-helix and random coil structures and increasing the content of β-sheet and β-turn structures in the protein secondary structure. This increased the proportion of structured secondary structures and decreased the proportion of structurally unstable random curly structures. As the proportion of L-Arg replacing NaCl increased, the degree of tertiary structure deconstruction of myosin increased, resulting in a decrease in FI. When it increased further (75 °C), myosin tail denaturation was involved in protein cross-linking. The disulfide bond effect exhibited a considerably greater in comparison to that of the control group (*p* < 0.05) while the contribution of hydrophobic interaction was constantly less than that of the control group (*p* < 0.05) throughout the heating process. Fluorescence detection results demonstrated that myosin could be fully unfolded by the combination of L-Arg with myosin. AFM results showed that when L-Arg is bound to myosin, the size consistency of myosin aggregates was improved, and the dispersion was more uniform than that of the control group. What is more, myosin aggregates evolved from small clusters to linear structures, and some myosin molecules were slightly assembled to form filaments, forming myosin aggregates dominated by short chains. Thus, myosin tended to form a good gel framework. Throughout the heating process, indicators such as turbidity, particle size, and AFM denoted that under the mediation of L-Arg, the solubility of myosin was improved, the folding behavior was fully carried out, and the excessive and disordered aggregation of the myosin head and tail was effectively inhibited.

Myosin formed a gel with a hydrophobic interaction and disulfide bond as the main forces. Additionally, as an alkaline amino acid, L-Arg could strengthen the hydrogen bond and electrostatic interaction of the gel and promote the transformation of the flaky-packed gel structure into an orderly, uniform, and dense three-dimensional network structure, thereby enhancing the gel’s ability to bind water and improving the elasticity and hardness of the gel. With the sodium-reduced system containing 0.3 M KCl, 0.1 M NaCl, and 0.2 M L-Arg, the low salt myosin gel exhibited good gel properties. However, the addition of L-Arg affects the sensory properties of low-salt beef products. Further research is needed to explore its specific mechanisms of action and regulation.

## 5. Conclusions

This study was intended to explore and compare the impacts of L-Arg, further replacing NaCl on the gelation characteristics, molecular structure, and aggregation behavior of beef myosin gels. L-Arg was found to increase the solubility of myosin, decrease turbidity, and induce conformational changes in the protein. The protein’s secondary structure underwent a transition from α-helix to β-sheet and β-turn structures. Meanwhile, a remarkable reduction occurred in the amount of random coiled structures. (*p* < 0.05). What is more, L-Arg improved the uniformity of myosin aggregate size, enhanced its dispersion, and inhibited the disordered aggregation of the myosin head and tail. Consequently, myosin aggregates transformed from small clusters into linear structures, forming thermal gels dominated by myosin oligomers and filaments. In the sodium-reduced system containing 0.3 M KCl, 0.1 M NaCl, and 0.2 M L-Arg, relatively uniform and regular myosin clusters were observed, which led to the formation of a gel with hydrophobic interactions and disulfide bonds as the predominant forces. Additionally, the beef myosin gel experienced a notable improvement in its hardness, elasticity, and WHC.

## Figures and Tables

**Table 1:** Effect of the L-Arg partial substitution of NaCl on the texture properties of myosin.

Groups	Hardness (g)	Springiness	Cohesiveness	Gumminess (g)
A	187.388 ± 13.116 ^d^	0.895 ± 0.004 ^d^	0.734 ± 0.005 ^d^	148.413 ± 8.524 ^e^
B	190.966 ± 12.457 ^c^	0.91 ± 0.002 ^c^	0.735 ± 0.003 ^d^	149.133 ± 9.699 ^e^
C	201.699 ± 11.425 ^b^	0.932 ± 0.003 ^b^	0.744 ± 0.006 ^c^	155.536 ± 9.939 ^d^
D	202.64 ± 9.811 ^b^	0.934 ± 0.006 ^b^	0.758 ± 0.005 ^b^	164.959 ± 10.118 ^c^
E	214.274 ± 11.519 ^a^	1.336 ± 0.003 ^a^	0.762 ± 0.003 ^a^	185.014 ± 7.118 ^a^
F	212.274 ± 11.425 ^a^	1.339 ± 0.002 ^a^	0.762 ± 0.005 ^a^	184.114 ± 10.628 ^a^
G	215.64 ± 9.811 ^a^	1.334 ± 0.004 ^a^	0.759 ± 0.005 ^ab^	180.413 ± 8.524 ^b^

Note: A (0.3 M NaCl), B (0.25 M NaCl + 0.05 M L-Arg), C (0.2 M NaCl + 0.1 M L-Arg), D (0.15 M NaCl + 0.15 M L-Arg), E (0.1 M NaCl + 0.2 M L-Arg), F (0.05 M NaCl + 0.25 M L-Arg), and G (0.3 M L-Arg). The results are expressed as M ± SD (n = 6). a–e indicates that there are significant differences (*p* < 0.05) in the mean of different letters in the same column.

**Table 2:** Effect of L-Arg partial substitution of NaCl on the tertiary structure of beef myosin during heating.

	Groups	Temperature						
		25 °C	35 °C	45 °C	55 °C	65 °C	75 °C	85 °C
λmax	T1	344 ± 0 ^Aa^	343.7 ± 1.2 ^Aa^	342.3 ± 0.6 ^Bb^	343.7 ± 1.2 ^Aa^	343 ± 0 ^Bab^	344 ± 0 ^Ba^	342.7 ± 0.6 ^Cab^
	T2	344 ± 1 ^Aa^	344.7 ± 0.6 ^Aa^	345 ± 0 ^Aa^	345 ± 0 ^Aa^	344.3 ± 0.6 ^Aa^	344.7 ± 0.6 ^Aba^	344.3 ± 0.6 ^Ba^
	T3	344.7 ± 0.6 ^Aa^	344.7 ± 0.6 ^Aa^	345 ± 0 ^Aa^	344.7 ± 0.6 ^Aa^	345 ± 1 ^Aa^	345.3 ± 0.6 ^Aa^	345.7 ± 0.6 ^Aa^
	T4	344.7 ± 0.6 ^Aa^	344.7 ± 0.6 ^Aa^	344.3 ± 0.6 ^Aa^	344.3 ± 0.6 ^Aa^	344.3 ± 0.6 ^Aa^	345 ± 0 ^Aa^	345.3 ± 0.6 ^Aba^
FImax	T1	282.4 ± 37.9 ^Ab^	258.7 ± 1.0 ^Ab^	287.0 ± 2.8 ^Ab^	358.3 ± 33.5 ^Aa^	371.6 ± 32.0 ^Aa^	361.9 ± 4.7 ^Aa^	360.9 ± 3.5 ^Aa^
	T2	214.4 ± 1.0 ^Bf^	217.5 ± 0.6 ^Be^	232.4 ± 0.6 ^Bd^	253.8 ± 1.9 ^Ba^	252.6 ± 0.8 ^Ba^	246.8 ± 0.5 ^Bb^	244.1 ± 1.2 ^Bc^
	T3	203.0 ± 0.8 ^Be^	206.8 ± 0.6 ^Dc^	209.9 ± 1.0 ^Cb^	216.3 ± 0.7 ^Ca^	215.7 ± 0.6 ^Ca^	209.9 ± 0.8 ^Cb^	204.6 ± 0.9 ^Cd^
	T4	209.0 ± 0.8 ^Bc^	211.8 ± 1.9 ^Cb^	212.3 ± 0.5 ^Cab^	214.3 ± 1.7 ^Ca^	213.9 ± 0.8 ^Cab^	209.4 ± 0.8 ^Cc^	200.7 ± 0.9 ^Dd^

Note: T1 (0.3 M KCl + 0.3 M NaCl), T2 (0.3 M KCl + 0.2 M NaCl + 0.1 M L-Arg), T3 (0.3 M KCl + 0.1 M NaCl + 0.2 M L-Arg), and T4 (0.3 M L-Arg). λmax is the maximum fluorescence emission wavelength and FImax is the maximum fluorescence intensity. Results: M ± SD (n = 3), A–D indicated that the mean values of different letters in the same column were significantly different (*p* < 0.05). a–f indicates that the mean values of different letters in the same row are significantly different (*p* < 0.05).

## Data Availability

The original contributions presented in the study are included in the article, further inquiries can be directed to the corresponding author.
